# Effect of Dietary *Lactobacillus plantarum* Supplementation on the Growth Performance, Intestinal Health, Antioxidant Capacity, and mTOR Signaling Pathway of Juvenile Coho Salmon (*Oncorhynchus kisutch*)

**DOI:** 10.3390/ijms26030907

**Published:** 2025-01-22

**Authors:** Qin Zhang, Lan Li, Rongxin Qin, Liuqing Meng, Dongsheng Liu, Tong Tong, Lixiao Xu, Yongqiang Liu, Weiguang Kong

**Affiliations:** 1 Guangxi Key Laboratory for Polysaccharide Materials and Modifications, Guangxi Marine Microbial Resources Industrialization Engineering Technology Research Center, School of Marine Sciences and Biotechnology, Guangxi Minzu University, 158 University Road, Nanning 530008, China; zhangqin@gxmzu.edu.cn (Q.Z.); lilan@stu.gxmzu.edu.cn (L.L.); rongxinqin@163.com (R.Q.); mengliuqing@stu.gxmzu.edu.cn (L.M.); liudongsheng@stu.gxmzu.edu.cn (D.L.); tongtong@gxmzu.edu.cn (T.T.); 2 Guangxi Institute for Drug Control, 9 Qinghu Road, Nanning 530023, China; 13397710823@163.com; 3 Key Laboratory of Breeding Biotechnology and Sustainable Aquaculture, Institute of Hydrobiology, Chinese Academy of Sciences, 7 Donghu South Road, Wuhan 430064, China

**Keywords:** coho salmon, *Lactobacillus plantarum*, growth performance, antioxidant capacity, mTOR signaling pathway

## Abstract

This study investigates the effect of dietary *Lactobacillus plantarum* supplementation on juvenile coho salmon (*Oncorhynchus kisutch*). Four groups of the juveniles (initial weight 103.87 ± 2.65 g) were fed for 10 weeks with four diets containing 0 (control diet), 10^5^ (T1), 10^7^ (T2), and 10^9^ (T3) cfu/g of *L. plantarum*. The main results are as follows: Compared with the control diet, the final weight, specific growth rate (SGR), and weight gain rate (WGR) of the juveniles fed the T1, T2, and T3 diet significantly (*p* < 0.05) increased, while the feed coefficient ratio (FCR) expressed an opposite trend. The activities of superoxide dismutase (SOD), catalase (CAT), and glutathione peroxidase (GSH-PX) in the serum of the juveniles fed the T2 diet significantly (*p* < 0.05) increased, while the malondialdehyde (MDA) expressed an opposite trend. The expression of phosphatidylinositol 4,5-bisphosphate 3-kinase (*pi3k*), AKT-interacting protein (*akt*), mechanistic target of rapamycin kinase (*mtor*), glucose-6-phosphate dehydrogenase (*g6pd*), *sod*, *cat*, and *gsh-px* genes in the liver of the juveniles fed the T2 diet significantly (*p* < 0.05) increased. In conclusion, the T2 diet significantly improved the growth performance, antioxidant capacity, and upregulated key mTOR pathway genes in juvenile coho salmon.

## 1. Introduction

Coho salmon (*Oncorhynchus kisutch*) is originally from the Pacific Ocean. Annually, approximately 120,000 metric tons of coho salmon are produced globally through aquaculture and wild catches, with around 80% of farmed coho salmon primarily found in Norway and Chile (FAO). However, this species does not have a natural distribution in China [1]. In recent years, to meet the growing nutritional needs of the people, coho salmon has been popularized in China and is one of the promising species for production [2]. Until now, there have been many studies on the nutritional requirements of coho salmon [3,4,5]; however, little research has been performed on feed additives that can promote growth in coho salmon. With the continuous increase in aquaculture density and the ongoing reduction in aquaculture space, the issue of diseases in aquaculture has become increasingly severe. Immunosuppression and disease outbreaks have caused significant economic losses for farmers [6]. To address these challenges, scientists and aquaculturists are placing great emphasis on developing cost-effective and environmentally friendly feed additives to promote the sustainable and healthy development of the aquaculture industry.

Probiotics are a type of live microorganism that are beneficial to the host, typically consisting of bacteria or yeast. They can promote the health of the host by regulating the gut microbiota, enhancing the immune system, and improving digestion and absorption [7]. Lactic acid bacteria (LAB) encompass a group of probiotics, including genera such as *Enterococcus*, *Lactobacillus*, *Lactococcus*, *Leuconostoc*, and *Weissella*. These Gram-positive bacteria primarily produce lactic acid as a major or sole byproduct of their fermentation metabolism. They are recognized as “generally regarded as safe” (GRAS) and are extensively utilized in aquaculture [8]. Previous research suggests that *Lactobacillus* spp. can attach to intestinal epithelial cells more quickly than pathogenic bacteria to compete for nutrients, thus inhibiting the growth of pathogenic bacteria [9]. Numerous studies have confirmed that LAB can significantly enhance growth [10], increase the activities of digestive enzymes, antioxidant enzymes, and lysozyme [11], improve intestinal flora and overall health [12], and boost resistance to infections by pathogenic bacteria in animals [13]. Furthermore, LAB is described as able to prevent soybean meal-induced enteritis in salmon [14], ferment multigrain meal in addition to salmon diets [15], and as a biological product to preserve salmon food [16]. *Lactobacillus plantarum* is a widely utilized strain of lactic acid bacteria (LAB) in the aquaculture industry. This bacterium offers numerous advantages to aquatic species, such as boosting immune responses, increasing resistance to diseases, and promoting better growth performance [13].

In this study, a lactic acid bacterium was isolated from the intestines of healthy coho salmon and identified as *L. plantarum*. Subsequently, various concentrations of *L. plantarum* were incorporated into the diets, and the relationship between *L. plantarum* and the growth of juvenile coho salmon was examined. This assessment was based on several factors, including growth performance, muscle composition, intestinal digestive enzyme activity, intestinal health, serum biochemical indices, antioxidant capacity, and the expression of genes associated with the PI3K/Akt/mTOR signaling pathway. Our findings aim to provide a theoretical foundation for the research and development of microecological preparations for coho salmon.

## 2. Results

### 2.1. Growth Performance

As shown in Table 1, the final weight, SGR, and WGR of juvenile coho salmon that were fed diets supplemented with different levels of *L. plantarum* were significantly higher (*p* < 0.05) compared to those fed the CK diet.

The FCR of juveniles fed the different levels of *L. plantarum* was significantly lower (*p* < 0.05) than that of juveniles fed the CK diet.

However, no significant difference (*p* > 0.05) was found in the CF, HSI, and SR of juveniles fed the different levels of *L. plantarum*.

### 2.2. Muscle Composition

As shown in Table 2, the content of muscle moisture of juveniles fed the T2 diet was significantly lower (*p* < 0.05) than that of juveniles fed the CK diet. The content of muscle moisture of juveniles fed the T1 and T2 diets was not significantly different (*p* > 0.05) from that of juveniles fed the CK diet.

The content of muscle crude protein of juveniles fed the T2 diet was significantly higher (*p* < 0.05) than that of juveniles fed the CK diet. The content of muscle crude protein of juvenile coho salmon fed the T1 and T2 diets was not significantly different (*p* > 0.05) from that of juveniles fed the CK diet.

However, no significant difference (*p* > 0.05) was found in the content of muscle ash and crude lipid of juveniles fed the different levels of *L. plantarum*.

### 2.3. Intestinal Tissue Morphology

**Figure 1:**
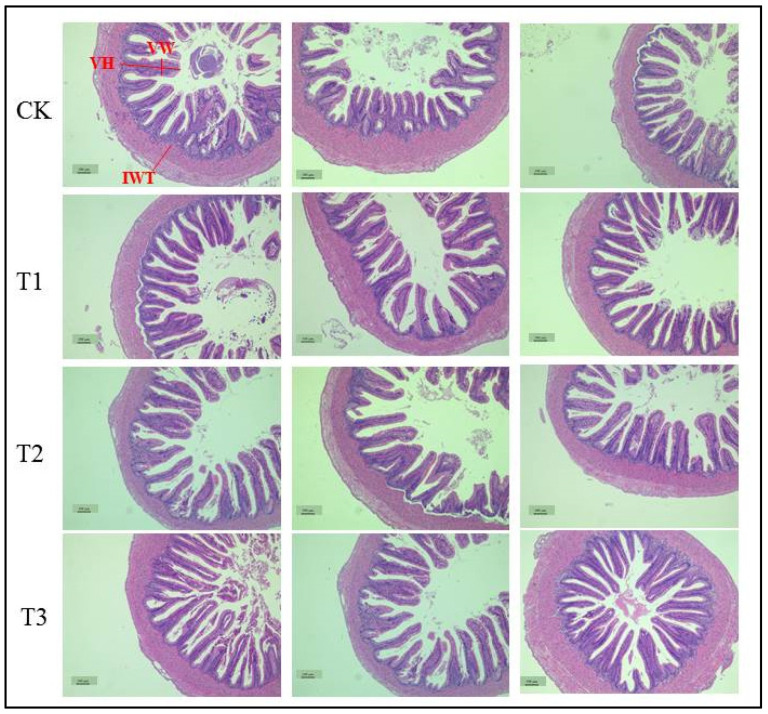
Effect of dietary *L. plantarum* supplementation on intestinal tissue morphology of juvenile coho salmon. VH means villi height, VW means villi width, and IWT means intestine wall thickness.

As shown in Table 3 and Figure 1, the VH in the intestinal region of juveniles fed the T2 and T3 diets was significantly higher (*p* < 0.05) than that of juveniles fed the CK diet. The VH in the intestinal region of juveniles fed the T1 diet was not significantly different (*p* > 0.05) from that of juveniles fed the CK diet.

However, no significant difference (*p* > 0.05) was found in the VW and IWT in the intestinal region of juveniles fed the different levels of *L. plantarum*.

### 2.4. Activity of the Intestinal Digestive Enzyme

As shown in Table 4, the activity of pepsin in the intestinal region of juveniles fed the T2 and T3 diets was significantly higher (*p* < 0.05) than that of juveniles fed the CK diet. The activity of pepsin in the intestinal region of juveniles fed the T1 diet was not significantly different (*p* > 0.05) from that of juveniles fed the CK diet.

The activity of α-amylase in the intestinal region of juveniles fed the T2 and T3 diets was significantly higher (*p* < 0.05) than that of juveniles fed the CK diet. The activity of α-amylase in the intestinal region of juveniles fed the T1 diet was not significantly different (*p* > 0.05) from that of juveniles fed the CK diet.

However, no significant difference (*p* > 0.05) was found in the activity of lipase in the intestinal region of juveniles fed the different levels of *L. plantarum*.

### 2.5. Serum Biochemical Index and Serum Antioxidant Capacity

As shown in Table 5, the content of GLU in the serum of juveniles fed the T2 and T3 diets was significantly lower (*p* < 0.05) than that of juveniles fed the CK diet. The content of GLU in the serum of juveniles fed the T1 diet was not significantly different (*p* > 0.05) from that of juveniles fed the CK diet.

The content of TP in the serum of juveniles fed the different levels of *L. plantarum* was significantly higher (*p* < 0.05) than that of juveniles fed the CK diet.

The activity of SOD in the serum of juveniles fed the T2 and T3 diets was significantly higher (*p* < 0.05) than that of juveniles fed the CK diet. The activity of SOD in the serum of juveniles fed the T1 diet was not significantly different (*p* > 0.05) from that of juveniles fed the CK diet.

The activity of CAT in the serum of juveniles fed the T2 and T3 diets was significantly higher (*p* < 0.05) than that of juveniles fed the CK diet. The activity of CAT in the serum of juveniles fed the T1 diet was not significantly different (*p* > 0.05) from that of juveniles fed the CK diet.

The activity of GSH-PX in the serum of juveniles fed the different levels of *L. plantarum* was significantly higher (*p* < 0.05) than that of juveniles fed the CK diet.

The content of MDA in the serum of juveniles fed the different levels of *L. plantarum* was significantly lower (*p* < 0.05) than that of juveniles fed the CK diet.

However, no significant difference (*p* > 0.05) was found in the content of TG and T-CHO and the activity of GOT and GPT in the serum of juveniles fed the different levels of *L. plantarum*.

### 2.6. Expression of pi3k, akt, mtor, g6pd, sod, cat, and gsh-px Gene

**Figure 2:**
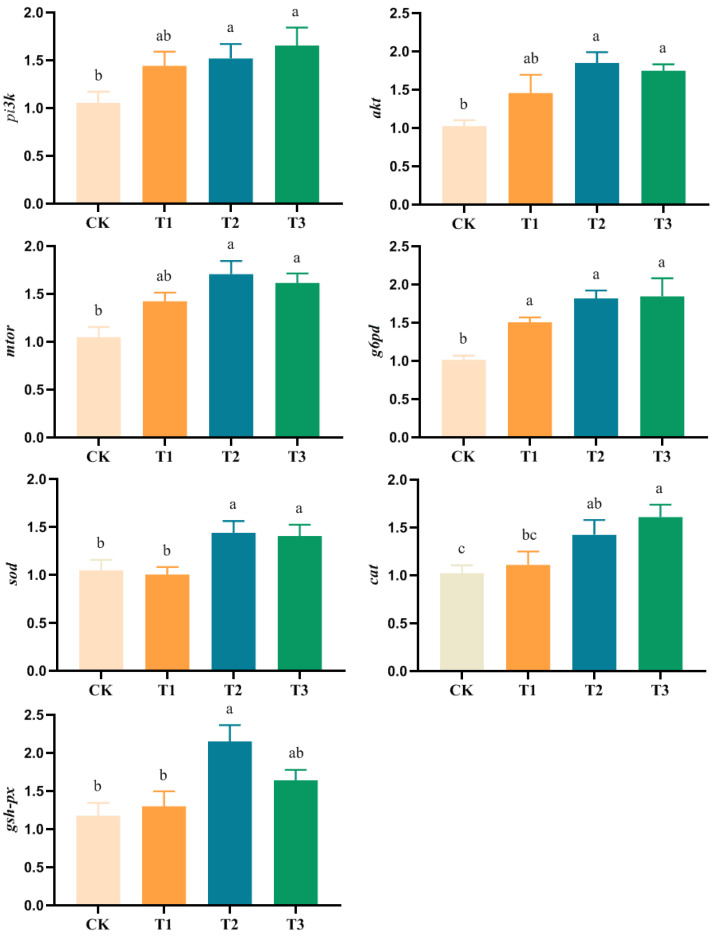
Effect of dietary *L. plantarum* supplementation on the expression of phosphatidylinositol 4,5-bisphosphate 3-kinase (*pi3k*), AKT-interacting protein (*akt*), mechanistic target of rapamycin kinase (*mtor*), glucose-6-phosphate dehydrogenase (*g6pd*), superoxide dismutase (*sod*), catalase (*cat*), and glutathione peroxidase (*gsh-px*) genes in the liver of juvenile coho salmon. All data are mean ± SE (*n* = 3), and in the same row, the values with different small letter superscripts are significantly different (*p* < 0.05).

As shown in Figure 2, the expression levels of *pi*3*k*, *akt*, *mtor*, *sod*, and *cat* genes in the liver of juveniles fed the T2 and T3 diets were significantly higher (*p* < 0.05) than those of juveniles fed the CK diet. The expression levels of *pi*3*k*, *akt*, *mtor*, *sod*, and *cat* genes in the liver of juveniles fed the T1 diet were not significantly different (*p* > 0.05) from those of juveniles fed the CK diet.

The expression level of *g6pd* gene in the liver of juveniles fed the different levels of *L. plantarum* was significantly higher (*p* < 0.05) than that of juveniles fed the CK diet.

The expression level of *gsh-px* gene in the liver of juveniles fed the T2 diet was significantly higher (*p* < 0.05) than that of juveniles fed the CK diet. The expression level of *gsh-px* gene in the liver of juveniles fed the T1 and T3 diets was not significantly different (*p* > 0.05) from that of juveniles fed the CK diet.

## 3. Discussion

Probiotics are primarily consumed by animals and travel through the esophagus to reach the intestine. Once there, they play a role in the host’s nutrient absorption, as well as contribute to immune function and metabolic processes. This, in turn, influences the growth and overall health of the animals [17]. The *L. plantarum* utilized in this study can establish itself within the intestinal environment. Feeding trials revealed that, compared to control diets, various levels of dietary *L. plantarum* supplementation significantly enhanced the growth performance of juvenile coho salmon. Nevertheless, an excessive amount of *L. plantarum* in the diet had an adverse effect on the growth of these fish. Overall, *L. plantarum* has demonstrated promising outcomes in aquaculture applications.

For instance, Son et al. [17] demonstrated that administering a diet supplemented with *L. plantarum* at a concentration of 10^8^ cfu/g for four weeks led to significant improvements in weight gain percentage, feed efficiency, lysozyme activity, and glutathione peroxidase activity in Spotted Grouper (*Epinephelus coioides*). This intervention also reduced mortality rates by over 20% following Streptococcus infection challenges. Yu et al. [18] observed that *L. plantarum* significantly boosted feed utilization, growth performance, and antioxidant capacity in Nile Tilapia (*Oreochromis niloticus*). Zheng et al. [19] noted that a cell-free extract of *L. plantarum* effectively enhanced the growth of White Shrimp (*Litopenaeus vannamei*) by improving digestive enzyme activities and increasing enterocyte height. On the beneficial side, LAB are rich in proteins, amino acids, and trace elements, which can be directly absorbed as nutrients. Additionally, LAB secrete organic acids and vitamins that help maintain intestinal ecological balance and stimulate growth [20,21]. Furthermore, Giorgia et al. [22] found that LAB could enhance feeding signals in Nile Tilapia by upregulating neuropeptide Y (NPY) and growth hormone-releasing peptide (ghrelin) while decreasing leptin levels, thereby promoting appetite and growth. Qin et al. [23] also discovered that LAB treatment in zebrafish (*Danio rerio*) resulted in increased weight gain, associated with the upregulation of growth-regulatory genes such as insulin-like growth factors (IGFs).

Research by Lee et al. [24] on Japanese eel (*Anguilla japonica*), Beck et al. [25] on olive flounder (*Paralichthys olivaceus*), and Zhang et al. [26] on Channel Catfish (*Ictalurus punctatus*) indicated that *L. plantarum* did not enhance the growth performance in these species. The diverse impacts of *L. plantarum* on various aquatic animals may be due to species-specific reactions and variations in the active compounds generated by different strains of *L. plantarum*. Moreover, Gao et al. [27] found that when LAB levels in the feed reached 10^7^ CFU/g, the growth and feed intake of *Haliotis discus hannai* tended to decline. This observation aligns with the notion that excessive LAB supplementation might not only suppress pathogenic microorganisms through competition for nutrients or attachment sites and antibiotic secretion but could also interfere with the normal growth and metabolism of beneficial microbes. Consequently, this may disrupt the intestinal microbial balance, leading to the overconsumption of limited nutrients without promoting animal growth. Therefore, it is crucial to carefully consider the concentration of probiotics included in diets [28].

In this study, when compared to a diet lacking *L. plantarum*, incorporating 10^7^ CFU/g of *L. plantarum* into the diet led to a significant increase in crude protein levels and a reduction in moisture content in the muscle tissue of juvenile coho salmon. This indicates that the nutritional condition of the juvenile coho salmon was enhanced. As the active substances contained in LAB were absorbed by the gastrointestinal mucosa, they could stimulate the secretion of a variety of digestive enzymes in the gastrointestinal mucosa. The secretion of digestive enzymes in the body can directly degrade the nutrients in the feed into easily absorbed small molecules of protein, small peptides, and amino acids, thereby promoting the digestion and absorption of nutrients, improving the nutritional value of the feed and the utilization rate of nutrients, improving the organization of the fish body, increasing the appetite of the fish, and reducing the feed coefficient. This improves the growth performance of the body and improves the composition of the muscles of fish [14]. The study by Mukherjee et al. [29] showed that using *Bacillus* can affect the body composition of Rohu (*Labeo rohita*). However, research showed that the addition of probiotics to the diet did not significantly affect the body composition of Olive flounder (*Paralichthys olivaceus*) [30] and Javanese carp (*Puntius gonionotus*) [31]. The reasons for the inconsistency of the above research results may be related to the different strains of probiotics, different types of fish, feeding environment, test period, and other factors. Further studies are needed to find out the reasons.

The intestine is an important organ for fish to digest and absorb nutrients in the feed, and pepsin, lipase, and α-amylase in the intestine are important digestive enzymes. So, the growth performance of animals is affected by intestinal digestive enzyme activity [32]. The results of this study showed that the pepsin of juvenile coho salmon increased significantly with the increase in the dietary *L. plantarum* supplementation. However, the lipase was not significantly different, which might be related to the ability of the LAB to break down the lipid. It was worth noticing that the α-amylase showed a downward trend, which may be due to the excessive consumption of intestinal carbon sources by the α-amylase. In conclusion, adding *L. plantarum* to the diet is beneficial to increase the digestive enzyme activity in fish. Similarly, more studies [33,34] have shown that not only LAB but other probiotics including yeast can improve digestive enzyme activity in animals. The reasons might be that, first, the colonization of LAB in the host intestine itself secretes or stimulates the host to secrete a variety of enzymes, thus improving the process of digestion and absorption. After colonizing the intestinal tract of animals, probiotics can secrete certain enzymes to the intestinal tract during the proliferation process, increase the enzyme content in the intestinal tract, and supplement the intestinal endogenous enzymes to a certain extent, thus improving the intestinal digestive enzyme activity of the body [35,36]. Second, the acidic metabolites of LAB reduce the pH of the animal’s intestine, and a lower pH environment may increase digestive enzyme activity. At the same time, the bioactive substances contained in probiotics can stimulate the digestive mucosa and promote the secretion of digestive enzymes, thus improving the activity of protease and lipase in the intestine and the digestibility of nutrients in the feed [37]. Third, because LAB could inhibit the growth of pathogenic bacteria in the intestinal tract, promote the growth of beneficial bacteria, regulate the balance of pathogenic bacteria and probiotics in the intestinal tract of fish, and effectively improve the microecological environment balance of the digestive tract of fish [8]. In addition, LAB improves the breeding environment and promotes animal feeding [38]. Some studies have also pointed out that one of the main modes of action and beneficial effects of LAB in aquaculture organisms is to supplement dehydrase, improve growth and feed efficiency, inhibit intestinal diseases, and improve intestinal function to enhance host nutrition [39]. LAB produces different enzymes in the digestive system, such as amylase, lipase, protease, and alkaline phosphatase, or stimulates the host’s digestive system to produce these enzymes [40]. LAB can also enhance the structure of intestinal epithelium, providing greater absorptive surface volume and improving nutrient absorption [41].

The uptake of nutrients is strongly correlated with intestinal structure [42]. The morphology of intestinal villi is considered to be a sign affecting the absorption capacity of aquatic animals [43]. The larger the surface area and density of intestinal villi, the more contact area there is between chyme and the intestine, and the more effectively the animal can absorb nutrients in the feed. The increase in IWT can increase the elasticity of the intestinal tract and promote the movement of feed in the intestine [44]. The results of this study show that compared with the CK diets, the diet that received 10^7^ CFU/g and 10^9^ CFU/g *L. plantarum* can significantly increase the VH of juvenile coho salmon, thus expanding the surface area of intestinal villi. It can be inferred that the improvement in the intestinal issue morphology of juvenile coho salmon is one of the reasons for promoting the growth of the juvenile. Similarly, adding functional feed additives or probiotics to feed can improve the intestinal villus surface area, thereby improving the intestinal morphology of animals and promoting animal growth [45]. Falcinelli et al. [46] showed that lactic acid bacteria can strengthen the intestinal epithelial structure. The addition of Lactobacillus rhamnosus increased the length of intestinal microvilli and intestinal epithelial cells, decreased the size of lipid droplets in the intestinal epithelium, increased the abundance of firmicutes, and decreased the abundance of actinomyces. The gut microbiome induces the radicals involved in cholesterol and triglyceride substitution, reduces the total cholesterol and triglyceride content in the body, increases the fatty acid level, and speeds up the growth of zebrafish [46]. However, at present, there is no exact study to clarify the relationship between probiotics and intestinal morphology and intestinal enzyme activity, and the exact process and mechanism of LAB-promoting fish growth still needs to be further studied and discovered.

The increase in total protein content in serum is beneficial to maintain osmotic pressure and improve the metabolism level and immunity [47]. Besides that, hematology can also indirectly reflect the general health status of animals after the consumption of functional feed additives [48]. In particular, when the liver is damaged or the liver function is weakened, cell membrane penetration increases, and the GOT, GPT, and T-CHO in the blood increase, thus leading to abnormal TG [49]. The results of this study show that compared with the CK diets, the diet that received 10^7^ CFU/g and 10^9^ CFU/g *L. plantarum* can significantly decrease GLU in the serum of juvenile coho salmon, increase TP, and there was no significant effect on liver health. These phenomena may be caused by the LAB in the digestive system using part of the glucose to ferment, which can break down indigestible proteins into small molecules that are easy to absorb [50]. Similar results of studies by Hou et al. [51] and Maniat et al. [52] showed that the dietary intake of LAB in animals could alleviate the stress response caused by postprandial hyperglycemia in their opinion. Some studies suggested that LAB may regulate colon flora, or other methods, to reduce T-CHO and TG in serum [53,54]. However, in this study, the decreasing trend of the TG and T-CHO was not significant, which needs to be explained by further studies. Interestingly, Dawood et al. [55] used heat-killed *L. plantarum* in diets, and they significantly increased GLU in serum of Tilapia, possibly because the heat-killed LAB lost their biological activity and could only be used as a dietary supplement in feed, thus increasing GLU in serum.

Reactive oxygen species (ROS) induce lipid peroxidation in cell membranes, resulting in the formation of malondialdehyde (MDA). Excessive MDA accumulation can cause cross-linking and polymerization of macromolecules like proteins and nucleic acids, thereby altering the structural and functional integrity of cell membranes. ROS are generated through two primary pathways: firstly, a minor amount is produced as a byproduct of normal cellular aerobic metabolism, and secondly, a significant quantity is generated by phagocytic cells during their antimicrobial activities [56]. Antioxidant enzymes, including superoxide dismutase (SOD), catalase (CAT), and glutathione peroxidase (GSH-PX), play a crucial role in detoxifying lipid peroxides, mitigating ROS-induced toxicity, and reducing MDA levels, thus safeguarding cellular health [57]. Therefore, higher antioxidant enzyme activity indicates that cells have a stronger ability to remove free radicals. The results of this study showed that after eating the probiotics, the antioxidant enzyme activity in the serum of juvenile coho salmon was significantly increased, while MDA was significantly decreased, which may mean that the antioxidant capacity of the fish was significantly improved. However, as mentioned above, excess LAB causes the excessive consumption of nutrients, resulting in a tendency to decrease the antioxidant enzyme activity. Many researchers have reported that LAB can improve the activity of antioxidant enzymes in the body or surface mucus of fish [58,59]. On the one hand, probiotics can produce a variety of metabolites with antioxidant capacity, such as niacin, extracellular polysaccharide, and butyric acid. These probiotics can increase folic acid content in animals and promote the synthesis of antioxidant enzymes in the body [60]. On the other hand, probiotics are thought to induce the gene expression of antioxidant enzymes that are released from the liver [61]. These behaviors have a positive effect on improving the antioxidant capacity of fish.

Finally, this study investigated the impact of *L. plantarum* on the digestive function and antioxidant capability of juvenile coho salmon at the molecular level. The phosphatidylinositol-3-kinase (PI3K)/protein kinase B (PKB/Akt)/mammalian target of rapamycin (mTOR) signaling pathway, which is classically associated with cellular growth and proliferation, was examined in this context. When amino acids are sufficient in the body, AKT promotes glucose absorption and mTOR is fully activated, directing the use of glucose for biosynthesis-related enzymes and nutrient storage [62,63]. Thus, the PI3K/Akt/mTOR signaling pathway is crucial for sustaining normal physiological functions and regulating growth and development [64]. In this study, the gene mRNA expression levels of the PI3K/Akt/mTOR pathway (*pi3k*, *akt*, and *mtor*), glucose-6-phosphate dehydrogenase (*g6pd*), and antioxidant enzymes (*sod*, *cat*, and *gsh-px*) were notably elevated in the T2 and T3 diets compared to the CK diets. This increase mirrors the trends observed in growth performance, digestive enzyme activity, and antioxidant enzyme activity. These changes may contribute to the faster growth rates seen in juvenile coho salmon fed diets supplemented with *L. plantarum*. This research has shown that LAB can activate the NRF 2 signaling pathway in mouse liver and upregulate the gene mRNA expression of antioxidant enzymes, such as *sod*, *cat*, and *gsh-px*, thus enhancing the antioxidant defense ability of the body [65]. Several studies have demonstrated that LAB can reduce the mRNA expression of stress response genes, including heat shock proteins, consequently improving overall growth performance [66]. Moreover, LAB could influence the intestinal barrier structure through modulating the mRNA expression levels of tight junction proteins [67]. To sum up, animals may be able to regulate their health by ingesting probiotics.

## 4. Materials and Methods

### 4.1. Experimental Diets and L. plantarum

*L. plantarum* was isolated from the intestines of healthy coho salmon by the authors in this study and was identified as *L. plantarum* by cluster analysis on the sequence of the 16S rRNA gene. The brief isolation processes were as follows: Intestinal contents from coho salmon were aseptically inoculated onto MRS AGAR medium (Shandong Tuopu Biological Engineering Co., Ltd., Yantai, China) and incubated at 37 °C for 12–24 h to observe colony morphology. Single colonies were selected using an inoculation loop, subjected to smear preparation and Gram staining, and examined under an optical microscope for morphological and Gram staining characteristics. Representative single colonies were subsequently inoculated into MRS liquid medium (Shandong Tuopu Biological Engineering Co., Ltd., Yantai, China) and cultured at 37 °C for 12 h. Genomic DNA was extracted using a bacterial genomic DNA extraction kit (Accurate Biology Biotechnology Engineering Ltd., Changsha, China) and used as the template for polymerase chain reaction (PCR) amplification of the 16S rRNA gene with universal primers 27F and 1492R. PCR products were analyzed by 1.0% agarose gel electrophoresis, and positive samples were sent to Sangon Biotech (Shanghai) Co., Ltd. (Shanghai, China) for sequencing. The sequencing data were compared against the National Center for Biotechnology Information (NCBI) database and identified as *L. plantarum*. The bacteria were cultured in de Man, Rogosa, and Sharpe Broth (MRS broth) for 12 h at 37 °C and collected by centrifugation at 5000× *g* for 10 min at 4 °C, and then they were washed twice with sterile saline (0.9%), and the concentration of the final suspension was adjusted to 10^10^ cfu/mL in sterile saline.

The experimental diets were supplied by Conkerun Ocean Technology Co., Ltd. in Weifang, China, as detailed in Table 6. All diets were processed into a fine powder with an 80-mesh size using a hammer mill (WF-20B, Zhenfeng Pharmaceutical Machinery, Jiangyin, China). The fine powder was then combined with four graded doses of *L. plantarum* in sterile saline and mixed in a roller mixer (CH-50, Zhenfeng Pharmaceutical Machinery, Jiangyin, China) for 15 min. Sterile distilled water (40% mass) was added to form a firm dough. This dough was manually divided into small balls, which were then sorted using meshes to select those with a diameter of approximately 2.50–3.55 mm. The resulting experimental diets consisted of four different concentrations of *L. plantarum*: CK (control diet, 0 cfu/g), T1 (10^5^ cfu/g), T2 (10^7^ cfu/g), and T3 (10^9^ cfu/g). All diets were dried using airflow at 30 °C until the moisture content fell below 100 g/kg, after which they were sealed in bags and stored at −20 °C for future use. To ensure the viability of *L. plantarum*, the experimental diets were prepared weekly.

The tolerance of washed *L. plantarum* cell suspensions to simulated gastric and small intestinal conditions was assessed using the following procedure. The 3 h survival rate in simulated gastric fluid (3-SR-SGF) and the 4 h survival rate in simulated intestinal fluid (4-SR-SIF) were determined according to the method described by Liu et al. [68]. Specifically, 0.2 mL of the washed cell suspension was added to a 2.0 mL screw-cap microfuge tube, followed by the addition of 1.0 mL of simulated gastric fluid (pH 2.0) or simulated intestinal fluid (pH 8.0), along with 0.3 mL of NaCl (0.5% *w*/*v*), resulting in a total volume of 1.5 mL. The mixture was vortexed at setting 5 for 10 s using a vortex mixer (SI-A256, Scientific Industries, New York, NY, USA) and incubated at 37 °C in a forced air-circulated incubator (Gallenkamp, Alchem Chemicals Ltd., Little Island, Co. Cork, Ireland). To evaluate gastric transit tolerance, 0.1 mL aliquots were taken after 3 h for the determination of total viable count. For the assessment of small intestinal transit tolerance, 0.1 mL aliquots were taken after 4 h for the same purpose.

Auto-aggregation (A-A) and cell surface hydrophobicity (CSH) were carried out by referring to the methods of Liu et al. [68]. In summary, to analyze the CHS, xylene was selected as an apolar solvent and mixed with a bacterial solution. The resulting two-phase system was vortexed thoroughly for 5 min. After incubating at room temperature for 1 h, the aqueous phase was removed, and its absorbance was measured at 600 nm (A_600nm_). For analyzing the A-A, the bacterial suspensions were incubated at 37 °C and monitored at its A_600nm_ after 24 h, and the A-A percentage was expressed as [1 − A upper suspension/A total bacterial suspension] × 100. The results are shown in Table 7.

The safety test of the *L. plantarum* referred to the method of Verschuere et al. [69]. Briefly, 120 juvenile coho salmon (103.87 ± 0.65 g) were immersed in *L. plantarum* suspensions with final concentrations of 0, 10^3^, 10^5^, and 10^7^ cfu/mL and three parallel groups of 10 fish per concentration of *L. plantarum*. After a complete water change every 24 h, the *L. plantarum* suspensions were re-added to each aquarium, and juvenile coho salmon signs and mortality were monitored for 7 days. Safety tests showed that *L. plantarum* did not cause symptoms and death of juvenile coho salmon (the survival rate was 100%).

The survival rate of *L. plantarum* in feed was determined by the living bacterium culture method [68]. The survival of the strain was obtained by culturing the target strain and using various media and conditions to detect the growth of the colony. The number of live cells per gram of *L. plantarum* in feed was calculated by counting the number of colonies growing in the plate. The experimental result showed that the survival rate of *L. plantarum* in the feed was 85.25–93.32%.

### 4.2. Culture and Sampling

Five hundred six-month-old juvenile coho salmon were sourced from a hatchery at the Benxi rainbow trout breeding farm in Liaoning, China. The use of these fish for the experiment was approved by the Ethics Committee of Guangxi Minzu University in Nanning, China (approval number: GXMZU 2021-003).

After disinfecting with potassium permanganate at a concentration of 1/100,000–1/50,000, the juveniles were acclimatized for 14 days. They were cultured under natural light (10 h:14 h) with water temperatures of 10–18 °C, a water intake of ≥100 L/s, surface velocity of ≥2 cm/s, dissolved oxygen levels of ≥6.0 mg/L, and a pH of 7.8–8.3. The juveniles were fed three times daily at 08:00, 12:00, and 16:00 with the CK diet, continuing until they showed no feeding behavior.

Following a 14-day acclimatization period, 240 juvenile coho salmon (initial average weight 103.87 ± 2.65 g) were randomly distributed into four groups, each replicated three times, resulting in a total of 12 net pens (dimensions: 1.0 × 1.0 × 0.8 m^3^, L × W × H), with 20 fish per pen. The juveniles were fed three times daily at 08:00, 12:00, and 16:00 for a duration of 10 weeks. Feeding was conducted to satiation, meaning food was provided until the fish no longer showed interest in feeding during each session. The rearing conditions throughout the experimental period mirrored those used during the acclimatization phase.

After a 10-week period, juvenile coho salmon were subjected to a 24 h fasting period and subsequently anesthetized using 40 mg/L of methanesulfonate 3-aminobenzoic acid ethyl ester (MS-222) supplied by Adamas Reagent, China. Nine fish from each net cage were randomly selected for sampling, with individual measurements were taken for body weight and length to assess the growth parameters. The anesthetized salmon were placed on a sterile, clean workspace to simplify the handling procedures. The tail vein region was sterilized by gently wiping it with a sterile cotton ball. Blood samples were collected from the caudal vein of the juvenile coho salmon using a sterile syringe and transferred into a 2 mL sterile enzyme-free centrifuge tube. The blood samples were then centrifuged at 3000× *g* for 15 min at 4 °C, with the resulting supernatant being identified as serum. Samples of dorsal muscle and foregut were separately collected and placed into sample bags. Liver samples were also collected, weighed, and stored in sample bags. All experimental samples were preserved at −80 °C for further analysis.

Furthermore, three additional fish from each net cage were randomly selected for intestinal health analysis, following the procedures outlined by Zhang et al. [7]. The anterior intestines were preserved in 4% formaldehyde, dehydrated using ethanol, embedded in paraffin, sectioned, and stained with hematoxylin–eosin. The intestinal villus height (VH), villus width (VW), and intestinal wall thickness (IWT) were examined and documented under an optical microscope.

### 4.3. Calculations and Analytical Methods

#### 4.3.1. Growth Performance

The specific growth rate (SGR), weight gain rate (WGR), feed coefficient ratio (FCR), condition factor (CF), hepatosomatic index (HSI), and survival rate (SR) were calculated using the following formula:

$$\mathrm{SGR}\;(\%)=100\;\times \;\frac{\ln\left(\mathrm{final}\;\mathrm{weight}\;(g)\right)-\;\ln\left(\mathrm{initial}\;\mathrm{weight}\;(g)\right)}{\mathrm{days}}$$

$$\mathrm{WGR}\;(\%)=100\;\times \;\frac{\mathrm{final}\;\mathrm{weight}\;(g)-\mathrm{initial}\;\mathrm{weight}\;(g)}{\mathrm{initial}\;\mathrm{weight}\;(g)}$$

$$\mathrm{FCR}=\frac{\mathrm{total}\;\mathrm{consumed}\;\mathrm{diet}\;\mathrm{weight}\;(g)}{\mathrm{final}\;\mathrm{weight}\;(g)-\mathrm{initial}\;\mathrm{weight}\;(g)}$$

$$\mathrm{CF}\;(\%)=100\;\times \;\frac{\mathrm{body}\;\mathrm{weight}\;(g)}{(\mathrm{body}\;\mathrm{length}\;{\left(\mathrm{cm})\right)}^{3}}$$

$$\mathrm{HSI}\;(\%)=100\;\times \;\frac{\mathrm{liver}\;\mathrm{weight}\;(g)}{\mathrm{body}\;\mathrm{weight}\;(g)}$$

$$\mathrm{SR}\;(\%)=100\;\times \;\frac{\mathrm{final}\;\mathrm{number}\;\mathrm{of}\;\mathrm{fish}}{\mathrm{init}i\mathrm{al}\;\mathrm{number}\;\mathrm{of}\;\mathrm{fish}}$$

#### 4.3.2. Muscle Composition Analysis

The muscle composition analysis was conducted according to the standard procedures outlined by the Association of Official Analytical Chemists (AOAC, 2005) [70]. To determine the moisture content, samples were dried in an oven at 105 °C until they reached a constant weight. The ash content was assessed by incineration in a muffle furnace at 550 °C. The crude protein content was measured using the Kjeldahl procedure, while the crude lipid content was analyzed via the Soxhlet extraction method.

#### 4.3.3. Determination of Serum Biochemical Index, Intestinal Digestive Enzyme Activity, and Antioxidant Capacity

The serum biochemical indicators, intestinal digestive enzyme activities, and antioxidant capacities were assessed using the methodologies outlined by Zhang et al. [7] and Liu et al. [68]. These parameters were evaluated with commercial kits supplied by the Nanjing Jiancheng Bioengineering Institute (Nanjing, China). The specific experimental principles and operational procedures were conducted according to the kit instructions, which are available for download at http://www.njjcbio.com (last accessed on 1 November 2024).

Pepsin was determined by the colorimetric method, and the unit of the pepsin activity in the intestinal was defined as follows: histone in intestinal supernatants breaks down protein to produce 1 μg of tyrosine per minute at 37 °C. The α-amylase was determined by the starch–iodine colorimetry method, and the unit of the α-amylase activity in the intestinal was defined as 10 mg of starch hydrolyzed by histone in intestinal supernatants at 37 °C for 30 min. The lipase was determined by the microplate method, and the unit of lipase activity in the intestinal was defined as 1 µmol substrate consumed per minute and per gram protein in intestinal supernatants at 37 °C.

The concentration of glucose (GLU) was measured using the glucose oxidase technique. The total cholesterol (T-CHO) levels were assessed through the cholesterol oxidase (COD-PAP) method. Triglyceride (TG) concentrations were evaluated by means of the glycerol phosphate oxidase (GPO-PAP) procedure. The total protein (TP) levels were quantified utilizing the Coomassie brilliant blue method. For GLU, T-CHO, and TG, the serum concentrations are expressed as the quantity per milliliter of serum, whereas the TP content in serum is specified as the amount per liter of serum. The aspartate aminotransferase (GOT) and alanine transaminase (GPT) were determined by the microplate method, and the unit of the GOT and GPT in the serum was defined as follows: the pyruvate is produced by the reaction of enzyme and substrate oxidizes NADH to NAD^+^ and causes the optical density (OD) 510 nm to decrease per 0.001 at 25 °C for 1 min.

Superoxide dismutase (SOD) was determined by the water-soluble tetrazole salt (WST-1) method, and the unit of SOD in the serum was defined as the amount of SOD per milliliter of serum when the SOD inhibition rate in the reaction solution reaches 50%. Catalase (CAT) was determined by the visible light method, and the unit of CAT in the serum was defined as the ability to decompose 1 µmol hydrogen peroxide (H_2_O_2_) per second and per milliliter of serum. Glutathione peroxidase (GSH-PX) was determined by the colorimetric method, and the unit of GSH-PX in the serum was defined as follows: the concentration of GSH-PX in the reaction system was reduced by 1 μmol/L when 0.1 mL serum was treated at 37 °C for 5 min, excluding the non-enzymatic reaction. Malondialdehyde (MDA) was determined by the thiobarbituric acid (TBA) method, and the unit of MDA in the serum was defined as the amount per mL of serum.

#### 4.3.4. Gene Expression in the Liver

To evaluate the expression levels of superoxide dismutase (*sod*), catalase (*cat*), glutathione peroxidase (*gsh-px*), glucose-6-phosphate dehydrogenase (*g*6*pd*), phosphatidylinositol 4, 5-bisphosphate 3-kinase (*pi*3*k*), AKT-interacting protein (*akt*), and the mechanistic target of rapamycin kinase (*mtor*) mRNA in the liver of juvenile coho salmon, we adopted the approach described by Liu et al. [71]. Specifically, total RNA was isolated from samples using the Steady Pure Universal RNA Extraction Kit, followed by reverse transcription into cDNA with the Evo M-MLV reverse transcription kit (both from Accurate Biology Biotechnology Engineering Ltd., Changsha, China). A quantity of 500 ng of total RNA was processed for each sample.

The quality and concentration of the extracted RNA were assessed via spectrophotometry, utilizing the absorbance ratio at 260:280 nm measured by an ND-2000 spectrophotometer (Thermo, Waltham, MA, USA). RNA integrity was confirmed through gel electrophoresis on a 1% (*w*/*v*) agarose TAE gel stained with Gel RedTM nucleic acid stain (UVP, Upland, CA, USA). Subsequently, 1 μg of total RNA was converted to cDNA using the RT Master Mix for qPCR provided by Takara Biotech Ltd. Co. (Dalian, China). The reverse transcription PCR protocol included one cycle at 50 °C for 30 min, followed by 95 °C for 5 min, and concluded with a hold at 5 °C for 5 min.

To conduct a real-time quantitative polymerase chain reaction (RT-qPCR), forward and reverse primers for the target genes were designed based on the genomic sequences of coho salmon retrieved from the NCBI database. These primers were synthesized by Sangon Biotech (Shanghai) Co., Ltd., located in Shanghai, China. The specific primer sequences are listed in Table 8. For normalization purposes, β-actin was selected as the stable reference gene.

The real-time quantitative PCR (RT-qPCR) analysis was performed using the LightCycler^®^ 96 system from Roche (Switzerland) and the SYBR Green Pro Taq HS-qPCR kit provided by Accurate Biology Biotechnology Engineering Ltd. (Changsha, China). The RT-qPCR protocol consisted of an initial denaturation step at 95 °C for 30 s, followed by 40 cycles of denaturation at 95 °C for 5 s and annealing/extension at 60 °C for 30 s. Relative gene expression levels were determined using the 2^−∆∆CT^ method [72].

### 4.4. Statistical Analysis

The data analysis was conducted using IBM SPSS Statistics 25 (Chicago, IL, USA). We performed a one-way ANOVA and evaluated the assumptions of normality and homogeneity of variance. In cases where significant differences were observed (*p* < 0.05), multiple comparisons were analyzed using Duncan’s test. Statistical results are presented as means ± standard error (SE).

## 5. Conclusions

In summary, these experimental findings indicate that supplementing the basal diet with an appropriate concentration of *L. plantarum* enhances the growth performance, intestinal digestive enzyme activities, serum biochemical indicators, and liver antioxidant capacity in juvenile coho salmon. Additionally, it upregulates the mRNA expression of genes involved in the PI3K/Akt/mTOR pathway. The most effective dietary concentration of *L. plantarum* for these improvements was found to be 10^7^ cfu/g.

## Figures and Tables

**Table 1:** Effect of dietary *L. plantarum* supplementation on the growth performance of juvenile coho salmon.

Index	CK	T1	T2	T3	F-Value	*p*-Value
Initial weight (g)	103.87 ± 2.65	103.87 ± 2.65	103.87 ± 2.65	103.87 ± 2.65	None	None
Final weight (g)	305.10 ± 4.27 ^b^	322.19 ± 4.23 ^a^	331.52 ± 4.11 ^a^	325.11 ± 3.67 ^a^	7.646	0.010
SGR ^1^ (%)	1.54 ± 0.02 ^b^	1.62 ± 0.02 ^a^	1.66 ± 0.02 ^a^	1.63 ± 0.02 ^a^	6.904	0.013
WGR ^2^ (%)	193.72 ± 4.11 ^b^	210.19 ± 4.08 ^a^	219.17 ± 3.96 ^a^	213.01 ± 3.53 ^a^	7.645	0.010
FCR ^3^	1.84 ± 0.04 ^a^	1.70 ± 0.03 ^b^	1.63 ± 0.03 ^b^	1.67 ± 0.03 ^b^	7.804	0.009
CF ^4^ (%)	0.92 ± 0.01	0.92 ± 0.00	0.93 ± 0.01	0.93 ± 0.00	0.275	0.842
HIS ^5^ (%)	1.25 ± 0.06	1.25 ± 0.04	1.26 ± 0.00	1.24 ± 0.05	0.023	0.995
SR ^6^ (%)	92.67 ± 1.67	95.00 ± 2.89	96.67 ± 1.67	93.33 ± 1.67	1.111	0.400

Note: All data are mean ± SE (*n* = 3), and in the same row, the values with different small letter superscripts are significantly different (*p* < 0.05). ^1^ SGR: specific growth rate. ^2^ WGR: weight gain rate. ^3^ FCR: feed coefficient ratio. ^4^ CF: condition factor. ^5^ HSI: hepatosomatic index. ^6^ SR: survival rate.

**Table 2:** Effect of dietary *L. plantarum* supplementation on muscle composition of juvenile coho salmon (%, dry weight).

Groups	CK	T1	T2	T3	F-Value	*p*-Value
Moisture (%)	72.86 ± 0.21 ^a^	72.22 ± 0.34 ^ab^	71.65 ± 0.18 ^b^	72.00 ± 0.35 ^b^	3.270	0.034
Crude protein (%)	71.10 ± 0.19 ^b^	71.13 ± 0.26 ^b^	71.85 ± 0.17 ^a^	71.57 ± 0.26 ^ab^	2.660	0.065
Ash (%)	4.65 ± 0.10	4.60 ± 0.06	4.62 ± 0.10	4.43 ± 0.08	1.423	0.254
Crude lipid (%)	19.90 ± 0.35	20.00 ± 0.29	20.02 ± 0.35	20.07 ± 0.22	0.056	0.982

Note: All data are mean ± SE (*n* = 3), and in the same row, the values with different small letter superscripts are significantly different (*p* < 0.05).

**Table 3:** Effect of dietary *L. plantarum* supplementation on intestinal tissue morphology of juvenile coho salmon.

Groups	CK	T1	T2	T3	F-Value	*p*-Value
VH ^1^ (μm)	406.03 ± 18.77 ^b^	439.37 ± 12.07 ^ab^	480.00 ± 9.99 ^a^	466.98 ± 22.79 ^a^	3.843	0.019
VW ^2^ (μm)	116.19 ± 6.29	107.62 ± 6.20	116.35 ± 10.02	100.19 ± 4.86	1.195	0.327
IWT ^3^ (μm)	214.29 ± 12.72	217.78 ± 11.16	205.40 ± 19.06	176.35 ± 14.00	1.672	0.193

Note: All data are mean ± SE (*n* = 3), and in the same row, the values with different small letter superscripts are significantly different (*p* < 0.05). ^1^ VH: villi height. ^2^ VW: villi width. ^3^ IWT: intestine wall thickness.

**Table 4:** Effect of dietary *L. plantarum* supplementation on the activity of the intestinal digestive enzyme of juvenile coho salmon.

Groups	CK	T1	T2	T3	F-Value	*p*-Value
Pepsin (U/mgprot)	36.92 ± 4.14 ^b^	38.05 ± 3.15 ^b^	50.51 ± 5.21 ^a^	50.10 ± 3.63 ^a^	3.269	0.034
α-amylase (U/mgprot)	1.28 ± 0.09 ^c^	1.52 ± 0.17 ^bc^	2.19 ± 0.13 ^a^	1.84 ± 0.14 ^ab^	8.510	0.000
Lipase (U/mgprot)	8.96 ± 0.19	9.57 ± 0.55	8.76 ± 0.45	9.42 ± 0.64	0.615	0.610

Note: All data are mean ± SE (*n* = 3), and in the same row, the values with different small letter superscripts are significantly different (*p* < 0.05).

**Table 5:** Effect of dietary *L. plantarum* supplementation on serum biochemical index and antioxidant capacity in serum of juvenile coho salmon.

Groups	CK	T1	T2	T3	F-Value	*p*-Value
GLU ^1^ (mmol/L)	4.37 ± 0.30 ^a^	4.35 ± 0.34 ^a^	3.51 ± 0.21 ^b^	3.38 ± 0.15 ^b^	4.172	0.013
TP ^2^ (mg/mL)	40.75 ± 3.49 ^c^	49.22 ± 2.86 ^b^	62.11 ± 5.78 ^a^	61.99 ± 4.38 ^a^	5.980	0.002
TG ^3^ (mmol/L)	2.07 ± 0.13	1.98 ± 0.17	1.87 ± 0.10	1.77 ± 0.17	0.805	0.501
T-CHO ^4^ (mmol/L)	10.51 ± 0.35	9.79 ± 0.40	9.17 ± 0.77	8.78 ± 0.65	1.755	0.176
GOT ^5^ (U/L)	3.93 ± 0.32	3.43 ± 0.35	3.05 ± 0.29	3.63 ± 0.17	1.608	0.207
GPT ^6^ (U/L)	3.63 ± 0.29	3.60 ± 0.40	3.99 ± 0.40	3.37 ± 0.53	0.386	0.763
SOD ^7^ (U/mL)	34.78 ± 2.44 ^c^	39.57 ± 2.48 ^bc^	44.72 ± 1.84 ^ab^	45.86 ± 1.04 ^a^	6.272	0.002
CAT ^8^ (U/mL)	51.70 ± 3.45 ^c^	56.20 ± 2.45 ^bc^	65.80 ± 2.95 ^a^	64.18 ± 3.01 ^ab^	5.019	0.006
GSH-PX ^9^ (U/mL)	476.62 ± 49.67 ^c^	681.99 ± 71.84 ^b^	989.10 ± 100.51 ^a^	837.36 ± 98.25 ^a^	7.063	0.001
MDA ^10^ (nmol/mL)	1.51 ± 0.04 ^a^	1.37 ± 0.04 ^b^	1.15 ± 0.06 ^c^	1.23 ± 0.05 ^c^	10.064	0.000

Note: All data are mean ± SE (*n* = 3), and in the same row, the values with different small letter superscripts are significantly different (*p* < 0.05). ^1^ GLU: glucose. ^2^ TP: total protein. ^3^ TG: triglyceride. ^4^ T-CHO: total cholesterol. ^5^ GOT: aspartate aminotransferase. ^6^ GPT: alanine transaminase. ^7^ SOD: superoxide dismutase. ^8^ CAT: catalase. ^9^ GSH-PX: glutathione peroxidase. ^10^ MDA: malondialdehyde.

**Table 6:** Experimental diet formula (g/kg of dried feed) and approximate composition (%, dry matter percentage).

Ingredient	Content
Fish meal	40.00
Chicken meal	5.00
Shrimp meal	5.00
Soybean meal	15.00
Peanut meal	7.00
Wheat middling	14.10
Starch	3.00
Fish oil	6.00
Soybean oil	2.50
Ca(H_2_PO_4_)_2_	1.00
Minerals premix ^a^	0.50
Vitamins premix ^b^	0.50
Choline chloride	0.30
Vitamin C	0.10
approximate composition	
Crude protein (%)	41.80
Crude lipid (%)	15.20
Ash (%)	7.30
Moisture (%)	9.50
Crude fiber (%)	3.40
Nitrogen-free extract (%)	22.80
Gross energy (MJ/kg)	18.50

Notes: ^a^. Composition (mg/kg mineral premix): AlK(SO_4_)_2_⋅12H_2_O, 123.7; CaCl_2_, 17,879.8;CuSO_4_⋅5H_2_O, 31.7; CoCl_2_⋅6H_2_O, 48.9; FeSO_4_⋅7H_2_O, 707.4; MgSO_4_⋅7H_2_O, 4316.8; MnSO_4_⋅4H_2_O, 31.1; ZnSO_4_⋅7H_2_O, 176.7; KCl, 1191.9; KI, 5.3; NaCl, 4934.5; Na_2_SeO_3_⋅H_2_O, 3.4; Ca(H_2_PO_4_)_2_⋅H_2_O, 12,457.0; KH_2_PO_4_, 9930.2. ^b^. Composition (IU or g/kg vitamin premix): retinal palmitate, 10,000 IU; cholecalciferol, 4000 IU; α-tocopherol, 75.0 IU; menadione, 22.0 g; thiamineHCl, 40.0 g; riboflavin, 30.0 g; D-calcium pantothenate, 150.0 g; pyridoxineHCl, 20.0 g; meso-inositol, 500.0 g; D-biotin, 1.0 g; folic acid, 15.0 g; ascorbic acid, 200.0 g; niacin, 300.0 g; cyanocobalamin, 0.3 g.

**Table 7:** Character of *L. plantarum.*

Index	3-SR-SGF ^1^	4-SR-SIF ^2^	A-A ^3^	CSH ^4^
Survival rate (%)	33.18 ± 2.08	41.93 ± 1.57	66.37 ± 0.95	25.50 ± 0.73

Note: ^1^ 3-SR-SRF: 3 h survival rate of simulated gastric fluid. ^2^ 4-SR-SIF: 4 h survival rate of simulated intestinal fluid. ^3^ A-A: auto-aggregation. ^4^ CSH: cell surface hydrophobicity.

**Table 8:** Forward and reverse primers of the genes for RT-qPCR.

Gene	Primer Sequence	Amplicon Size (bp)	Gene Bank
*β-actin*	F: TGACCCAGATCATGTTTGAGACC	146	XM_031811226.1
	R: CTCGTAGATGGGTACTGTGTGGG		
*pi*3*k* ^1^	F: CCAGTGGCTCAAGGACAAGAACAG	98	XM_020466892.2
	R: GGATGAAGGTGGCTACGCAGTATC		
*akt* ^2^	F: GCAGCCATCCTACAATC	178	XM_031831237.1
	R: TGAAACAGGGTCCACAAG		
*mtor* ^3^	F: CTTCGCCAACTACCTCCG	139	XM_020506200.2
	R: TGCCCTTCACCTCAAACT		
*g*6*pd* ^4^	F: GGCTGAGCGGTTGTCTGTGTTC	90	XM_031793979.1
	R: TGGGTGTGGAGGTTGGAGAAGG		
*sod* ^5^	F: GGGAGCCTGCTACATGGTAATGC	108	XM_020497014.2
	R: CCTTCTTCTCTGCTGTCGATGATGG		
*cat* ^6^	F: CACCTATCGCCGTCCGCTTC	110	XM_020456233.2
	R: CCAGTTGCCCTCGTCAGTGTAG		
*gsh-px* ^7^	F: ACCAACACCCACCCTGTCTTTG	127	XM_020487159.2
	R: AAGAGATGTCTGTCCTGCTGATGG		

Note: F: forward primer. R: reverse primer. *β-actin* was the non-regulated reference gene. ^1^ *pi3k*: phosphatidylinositol 4,5-bisphosphate 3-kinase. ^2^ *akt*: AKT-interacting protein. ^3^ *mtor*: mechanistic target of rapamycin kinase. ^4^ *g6pd*: glucose-6-phosphate dehydrogenase. ^5^ *sod*: superoxide dismutase. ^6^ *cat*: catalase. ^7^ *gsh-px*: glutathione peroxidase.

## Data Availability

Data will be made available on request.
